# Denaturation of Water in Alkaline Melts

**DOI:** 10.1002/chem.202502562

**Published:** 2025-12-03

**Authors:** Xudong Zhang, Oldamur Hollóczki, Johannes Ingenmey, Barbara Kirchner, Michael Ruck

**Affiliations:** ^1^ Faculty of Chemistry and Food Chemistry TUD Dresden University of Technology Dresden Germany; ^2^ Faculty of Science and Technology, Department of Physical Chemistry University of Debrecen Debrecen Hungary; ^3^ Mulliken Center for Theoretical Chemistry University of Bonn Bonn Germany

**Keywords:** ab initio molecular‐dynamics simulation, alkali, hydroflux, vapor pressure, water

## Abstract

Extremely high base concentrations (*c*_B_) in ultra‐alkaline liquids, also known as hydroflux, alter the thermodynamic and structural properties of water. Mixtures of water and alkali (*A*OH, *A* = Na, K) with molar base ratios of *q*(*A*) = *n*(H_2_O):*n*(*A*OH) ≤ 2:1 (*c*_B_ ≥ 25 mol L^−1^) show an overproportionally reduced vapor pressure compared to more diluted systems. The vapor pressure of a melt with *q*(*A*) = 0.8 (*c*_B_ = 70 mol L^−1^) at 200°C is negligible. Ab initio molecular dynamics simulations revealed substantial structural reorganization of the hydrogen bonding network in the equimolar mixture of KOH and water. Distinctive molecular features included altered coordination geometries, shortened hydrogen bonds, and frequent proton transfer events, including Grotthuss diffusion, indicative of an altered hydrogen‐bond network and increased proton mobility. Cluster population analysis shows that a significant number of H_3_O_2_^−^ anions are present, which exhibit a near symmetrical hydrogen bond with O···H distances <1.28 Å. The hydroflux can be seen as an intermediate between an alkaline solution and a molten salt {K^+^·H^+^·2OH–}, in which the water has a vanishing chemical activity.

## Graphical abstract

Vapor pressure measurements and ab initio molecular‐dynamics simulations reveal that in ultra‐alkaline media with base concentrations up to 70 mol L^−1^, the activity of water is dramatically reduced by strong bonding interactions. These disrupt traditional hydrogen bonding patterns, alter vapor‐liquid equilibria, and significantly change molecular coordination and proton dynamics.

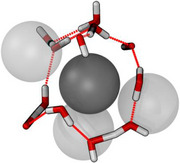

## 1 Introduction

To increase the sustainability of chemical industries, alternative reaction pathways are being sought that require less energy, are more atomically efficient, and produce less waste overall. New approaches include chemical reactions using the hydroflux method. A hydroflux medium can be considered a highly concentrated aqueous electrolyte solution or the melt of a wet salt. Typical molar ratios of water to salt *q*(*A*) = *n*(H_2_O):*n*(*A*OH) are in the range from 0.8 to 2, which corresponds to solutions with base concentrations 70 ≤ *c*_B_ ≤ 25 mol L^−1^ (molar, m). The addition of water to salts lowers the melting point. For example, pure KOH melts at 360°C, KOH·H_2_O at 127°C.[1] Syntheses in a hydroflux medium are characterized by moderate temperatures of up to 240°C (limited by the autoclave material) and short reaction times (often less than one day). Typically, the products are single‐phase, well crystallized, and obtained in high yields. This approach enables the synthesis of known substances with greater efficiency compared to conventional methods but, also facilitates the discovery of new compounds. The term “hydroflux” has been coined in 1975 by *Elwell* and *Scheel*, who also suggested using hydroflux as a reaction medium.[2] This was however only done once by *Harms* and *Gunßer* in 1986, who employed a hydroflux medium based on NaNO_3_ and KNO_3_ to synthesize gallium oxides.[3] In the last decade, first *zur Loye* [4, 5, 6, 7, 8] and then we [9, 10, 11, 12, 13] took up this idea again and applied it to synthesize a wide variety of oxometalates in highly concentrated alkaline media. Lately, the method has attracted increasing scientific attention.[14, 15, 16, 17]

In the course of these investigations, it became clear that alkaline hydroflux is a unique reaction medium. Firstly, the vapor pressure appeared to be much lower than in more water‐rich solutions, which are used in, for instance, hydrothermal synthesis.[4, 6] Secondly, despite the aqueous solvent system, highly hygroscopic compounds were also possible to obtain.[11] Thirdly, the ultra‐alkaline medium is far from standard conditions, so the tabulated electrochemical potentials do not apply, resulting in unexpected redox chemistry.[11, 13]

Since the unique physicochemical features of hydroflux media should be the key to their efficiency and to obtain peculiar materials in the syntheses performed in them, understanding and characterizing the properties of these solvents is essential to their further development, and to achieve their full potential. Although the behavior of electrolytes and alkaline solutions is textbook chemistry, knowledge becomes scarcer when approaching toward high concentrations, and therefore, further in‐depth studies are necessary in this field.

The lack of information is especially apparent for molecular‐level theoretical studies, which so far have focused on less concentrated systems. Besides the influence of the electronic structure theory and the effect of nuclear quantum effects,[18, 19] the state of the hydronium [20] or hydroxide ions [18, 21] in water is highly debated, even regarding the better investigated systems, and no final arguments seem to have been made. For the hydronium ion, several schools of thought are present, including the idea of the Zundel ion H_5_O_2_^+^ and the Eigen ion H_9_O_4_^+^, with a proton equally shared between two water molecules in the former and a H_3_O^+^ ion surrounded by three water molecules in the latter case.[20] Whereas *Voth* shows strong arguments in favor of the solvation of the hydronium ion as a distorted Eigen ion,[22] some studies suggest a mixture of these two, in which one hydronium ion and one of the three water molecules transiently become a Zundel‐like structure within the Eigen ion as a special pair dance.[18, 20] *Hassanali* highlighted another aspect, namely, for the recombination of hydronium separated by two water molecules from the hydroxide, a compressed wire effect comes into play, making all involved (hydrogen) bonds shorter than in the normal water system.[23]

For the solvated hydroxide ion, a tetrahedral (accepting three hydrogen bonds and donating one) and a hypercoordinated (accepting four hydrogen bonds and donating none) solvation mode have been shown to be present.[18] Both the solvated hydronium and tetrahedral hydroxide ions can rearrange in aqueous solutions via proton transfers to or from the surrounding water molecules. Through these proton transfer events, the charge can diffuse faster in solution than the actual atoms, which is referred to as structural or Grotthuss‐type diffusion [24] in the literature. Although the charge diffuses in both systems (i.e., in either acidic or basic media), hydroxide ions can, interestingly, contribute to conductivities remarkably less than hydronium ions. The reason for this remarkable discrepancy was suggested to be the high stability of the hypercoordinated hydroxide ion, which can, therefore, be considered a resting state of the structural diffusion.[18]

In light of the aforementioned various aspects of ion solvation and Grotthuss diffusion, the question is apparent: what kind of molecular structure and dynamics arise from these solvation modes and diffusion mechanisms in highly concentrated alkaline solutions? It is very likely that the unusual reactions that occur in hydroflux media are due to unique behavior at the molecular level, and therefore, understanding it in detail is crucial. This prompted us to investigate the reduced chemical activity of water in alkaline hydroflux media more thoroughly. The highly aggressive nature of the ultra‐alkaline medium, which increases when heated, requires a reaction vessel that does not allow optical or spectroscopic examination with simple means. We therefore performed vapor pressure measurements in a polytetrafluoroethylene (PTFE) lined autoclave as well as ab initio molecular‐dynamics simulations (AIMD). Together with the earlier experimental results and the compounds crystallized from alkaline hydroflux in recent years, a coherent picture emerges.

## 2 Results and Discussion

### 2.1 Vapor Pressure

The pressure in a PTFE‐lined steel autoclave equipped with an automated manometer (Figure S1 of the Supporting Information) was monitored (Figure S2) for different base concentrations *q*(*A*) = *n*(H_2_O):*n*(*A*OH) for *A* = Na, K between room temperature and 200 °C. For this purpose, solid NaOH or KOH and water were filled into the autoclave under ambient conditions. The heating time to 200 °C was set to 16 h, which corresponds to a rate of slightly less than 0.5 K min^−1^. The autoclave was kept at 200 °C for 6 h and then cooled to room temperature within 2 h.

The manufacturer specifies an accuracy of 1 K for the temperature sensor and 2 bar for the pressure sensor, which can measure up to 200 bar. To check for nonequilibrium effects in the first heating cycle, the experiment was repeated after 30 min using the same heating rate. We found that the *p*/*T* data for subsequent heating curves agree within experimental accuracy (Figure S3 and S7, Table S2), indicating that equilibrium was established during the measurement. Even at the highest base concentrations, we found no residues of solid NaOH or KOH after the measurements, so that the composition of the molten hydroflux medium should correspond to the ratio of the starting materials. Consequently, the (unknown) melting point of the mixtures with very high alkali content must also lie below 200 °C in all cases.

**FIGURE 1:**
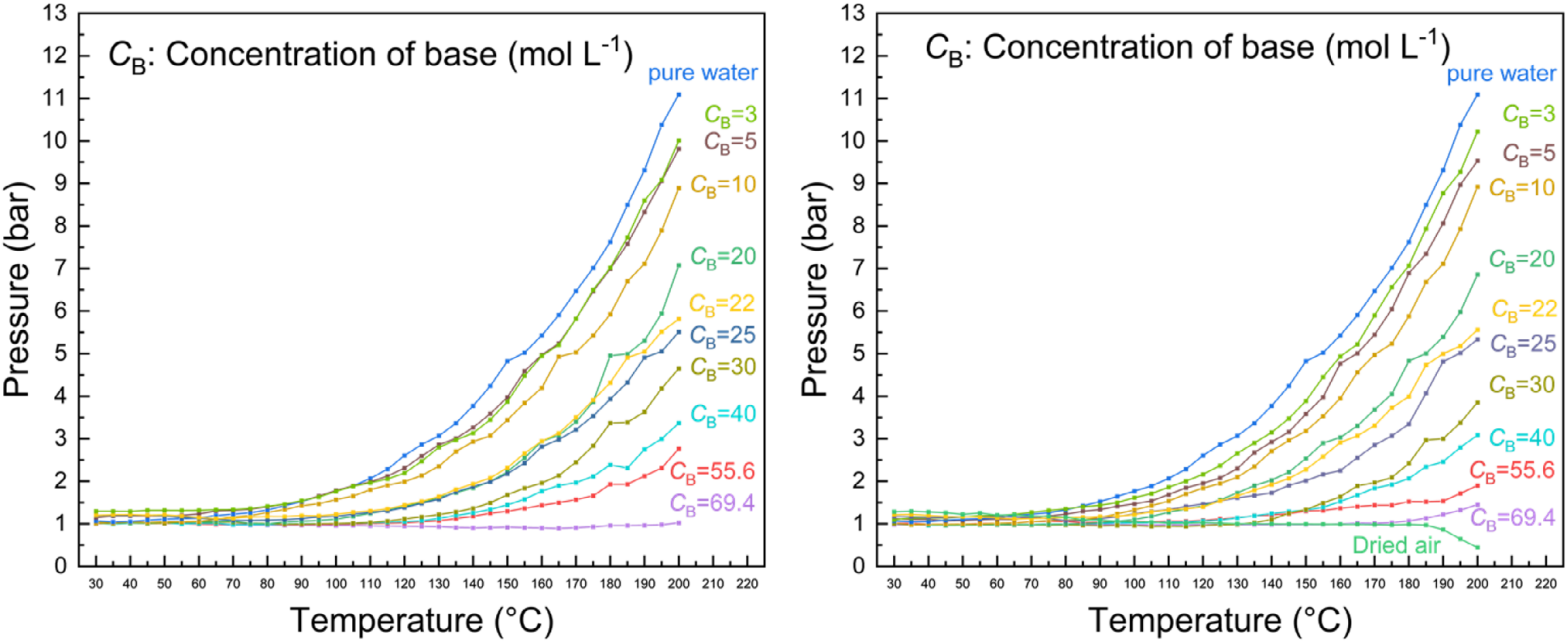
Total pressure developed in a PTFE‐lined autoclave when heating mixtures of water and solid *A*OH (left: *A* = Na; right: *A* = K) with different base concentrations.

Figure 1 and Table 1 show the temperature‐dependent evolution of the measured total pressure in the autoclave for mixtures of alkali *A*OH (*A* = Na, K) and water with various concentrations. Up to the highest concentration of almost 70 m, that is, a molar water‐base ratio of *q*(*A*) = 0.8, the vapor pressure decreases, and the estimated boiling point increases with increasing base concentration. The same observation has been made earlier for NaOH and KOH solutions with concentrations up to 25 and 18 m, respectively.[25] For *q*(*A*) ≤ 1, which nominally corresponds to a concentration of ≥55.6 m, a vapor pressure exceeding 1 bar is observed only close to or above 200 °C. This confirms previous reports of a remarkably low vapor pressure that develops during hydroflux syntheses. [4, 6] It also resembles the behavior of ionic liquids.[26]

**TABLE 1:** Total pressure in the autoclave after heating *A*OH solutions with various concentrations to 150°C and 200°C. The boiling point *T*_b_ is here defined as the temperature at which the total pressure exceeds its room temperature value during heating. First value *A* = Na, second value in italics *A* = K.

*q*(*A*)	*c*_B_ (mol L^−1^)	$x_{H_{2}O}$	*p* (bar) at 150°C	*p* (bar) at 200°C	*T*_b_ (°C)
0.8:1	69.4	0.44	*0.92 / 0.98*	*1.02 / 1.45*	*>200 / >200*
1:1	55.6	0.50	*1.29 / 1.29*	*2.77 / 1.90*	*187 / >200*
1.39:1	40	0.58	*1.44 / 1.32*	*3.37 / 3.09*	*171 / 177*
1.87:1	30	0.65	*1.68 / 1.33*	*4.65 / 3.86*	*161 / 171*
2.23:1	25	0.69	*2.17 / 2.02*	*5.51 / 5.04*	*145 / 150*
2.53:1	22	0.72	*2.31 / 2.28*	*5.81 / 5.36*	*142 / 143*
2.78:1	20	0.74	*2.22 / 2.53*	*7.08 / 6.86*	*145 / 139*
5.56:1	10	0.85	*3.44 / 3.18*	*8.89 / 8.92*	*120 / 127*
11.1:1	5	0.92	*3.98 / 3.58*	*9.81 / 9.53*	*110 / 122*
18.7:1	3	0.95	*3.87 / 3.88*	*10.01 / 10.22*	*112 / 115*
Pure water	0	1.00	4.82	11.09	107

For the samples with base concentrations *c*_B_ ≥ 30 m, a slight decrease of the total pressure is observed upon heating (Figure 1). This is unexpected, since at least the increase in the gas pressure of the enclosed air should be seen. A reduction of the pressure below the value at room temperature can be rationalized if the solution/melt has a higher density than the weighted average of the components. Additional small contributions could arise from the absorption of the CO_2_ content of the enclosed air and some oxygen that reacts with the alkaline medium to form superoxide and peroxide ions. The physisorption of molecular oxygen is negligible in aqueous alkaline solutions.[27] However, the observed effects, especially in the upper temperature range, are much too large to be explained in this way.

As long as there is a liquid phase, the vapor pressure should be independent of the filling level of the autoclave. Using the ideal gas law to estimate the amount of water in the gas phase suggests that there should be enough water in all experiments to ensure the presence of a liquid phase at all temperatures. However, we found an unexpected dependence of the measured pressure of pure water on the filling level, as well as negative deviations from the theoretically expected vapor pressure of water (about 15.5 bar at 200°C [25]). Residual impurities in the “deionized” water used were not responsible for this effect, as an experiment with freshly distilled water showed (Figure S6).

Finally, a measurement with the autoclave containing only dry air did not show the expected linear increase in internal pressure with temperature. Instead, a slight decrease was found, which grew dramatically above 190°C (Figure 1, right). Such a behavior can be caused by a combination of trapped air inside the encapsulated pressure sensor, resulting in pressure compensation, and leaks in the autoclave that reduce the air pressure in the autoclave volume but not inside the pressure sensor. The leakage rate cannot be high, as the escape of hot gas to the outside was not perceptible, and the pressure built up to 11.5 bar.

**FIGURE 2:**
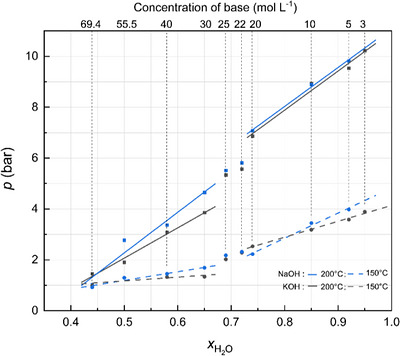
Relationship between the total pressure in the autoclave and the mole fraction of water in *A*OH solutions and melts (*A* = Na, K) at 150 °C and 200 °C.

Since the pressures measured with this device cannot be regarded as correct absolute values for the total pressure, no further corrections were applied to the measured data. Nevertheless, the measurements that were carried out with the same amount of water should allow for relative comparisons. The plots of the pressure at 150 °C and 200 °C against the mole fraction of water $x_{H_{2}O}$ shows two separated regimes for both alkali, each with almost linear dependency (Figure 2). In the range 0.5 ≤ $x_{H_{2}O}$ ≤ 0.65, which corresponds to the highly concentrated solutions 55.6 m ≥ *c*_B_ ≥ 30 m, the vapor pressure of KOH solutions is 1 to 2 bar lower than would be expected from a linear extrapolation of the range of the less concentrated solutions with $x_{H_{2}O}$ ≥ 0.74, that is, *c*_B_ ≤ 20 m. The difference between the two regimes is more pronounced for KOH than for NaOH. In between the two regimes lies the base concentration *c*_B_ = 25 mol L^−1^. Therefore, we suggest using this value to distinguish between hydroflux and hydrothermal conditions in aqueous alkaline media.

**FIGURE 3:**
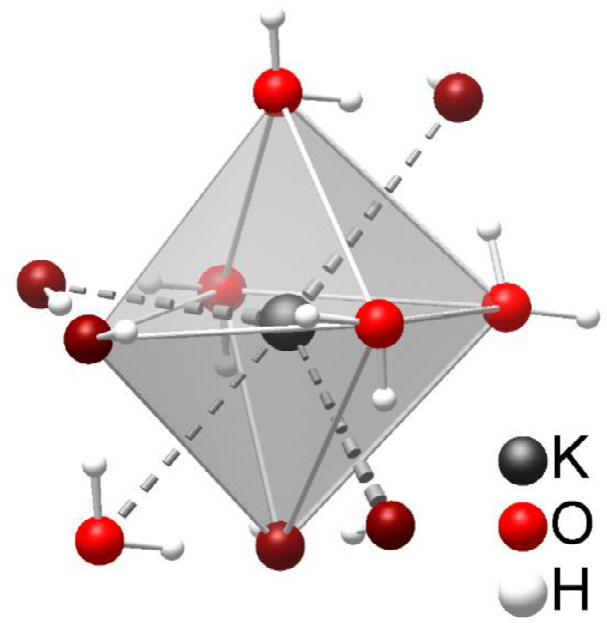
[6+4]‐coordination of the K^+^ cation in solid KOH·H_2_O. The light red colored oxygen atoms belong to the water molecules, the dark red colored ones to the hydroxide ions. K–O distances in the distorted octahedron range from 279 to 294 pm, the dotted connections range from 327 to 362 pm. The distances to the next two O atoms (not shown) are 429 and 432 pm, which exceed the distance to the next K^+^ cation (394 pm).

The corresponding molar water‐base ratio of about *q*(*A*) = 2 translates this threshold concentration into a molecular picture: The special properties of alkaline hydroflux occur when there is no more than one water molecule per ion. To rationalize these observations, it is necessary to understand the structure and intermolecular interactions within the system and the difference from a dilute aqueous solution. The typical coordination of a potassium cation, as observed in crystals, involves six water molecules or hydroxide ions. [K(OH)(H_2_O)_5_] groups without additional free water molecules are nominally obtained at *q*(K) = 5. A higher base concentration results in condensation of these complexes with bridging water and hydroxide ligands. In agreement, K^+^ cations in solid KOH·H_2_O, which could be seen as a crystallized hydroflux with *q*(K) = 1, have octahedral coordination (Figure 3) and every oxygen atom coordinates on average three K^+^ cations.[1] Such strongly bonded water molecules develop a very low vapor pressure. On the other hand, the water molecules reduce the electrostatic interactions between K^+^ and OH– ions, thus lowering the melting point compared to anhydrous KOH.

### 2.2 Ab Initio Molecular‐Dynamics Simulations

To understand the molecular‐level effects that lead to the aforementioned properties of hydroflux media, we performed *ab initio* molecular dynamics simulations on a system containing 150 H_2_O molecules and 150 K^+^ cations and OH– anions, corresponding to *q*(K) = 1 or *c*_B_ = 55.6 m. For the simulation details, see the Supporting Information. The visual inspection of the trajectory showed that within the 50‐ps physical time of the simulation, numerous proton transfer processes occurred, suggesting that this system is highly dynamic. This finding is in clear contrast with earlier studies for dilute systems, in which such proton transfers were found significantly rarer. To support this observation with quantitative data, the structure and dynamics of the system were analyzed in detail.

**FIGURE 4:**
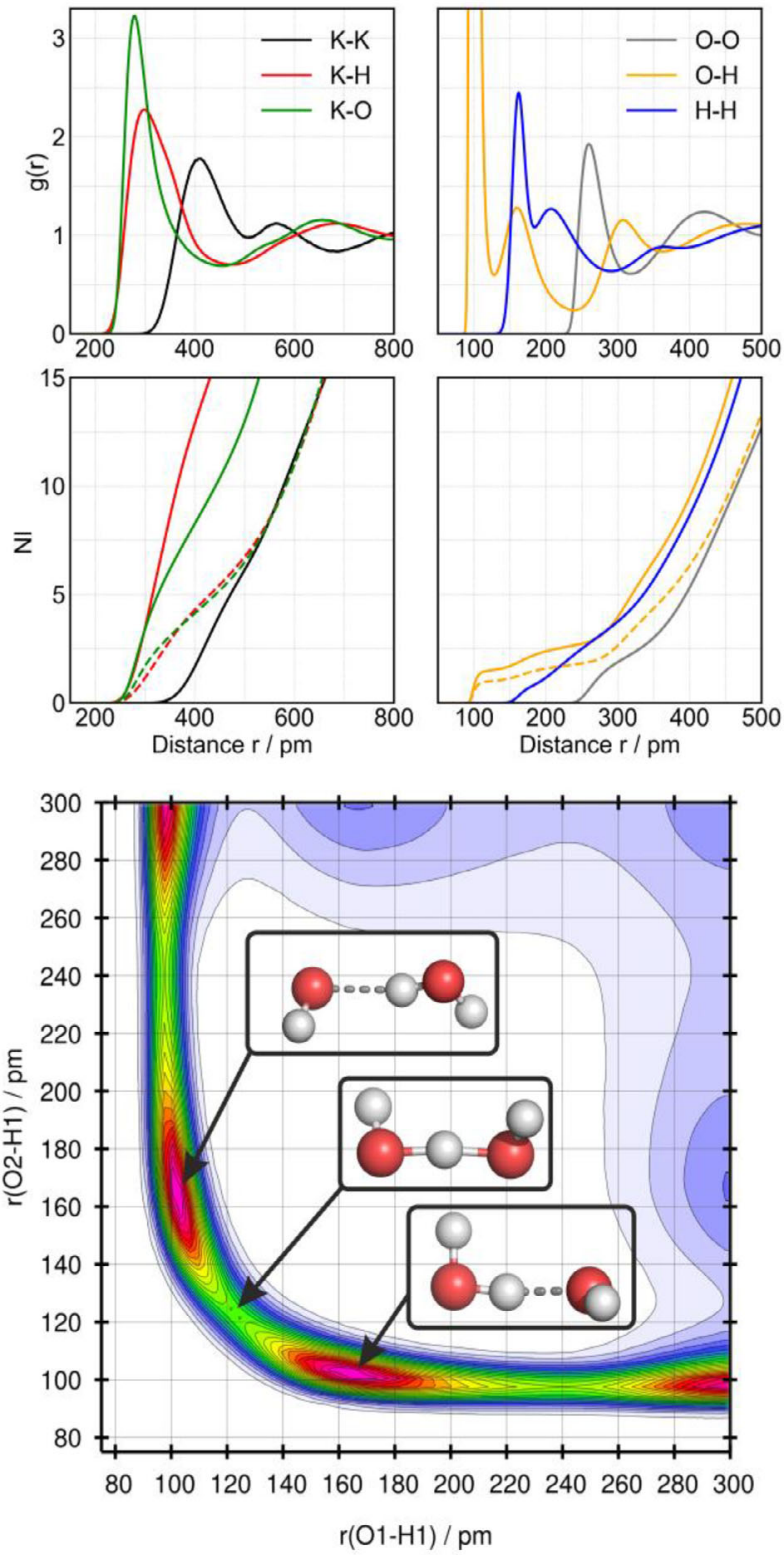
Upper panel: Radial distribution function (above) and number integral (below) of left: K atom with K (black), H (red), and O (green) atoms; and right: O atom with O (dark grey), H (orange), and H with H (blue) atoms. Note the x‐axis is different on the left than on the right. The dotted lines are the reversed coordination in the case of mixed atom functions. Lower panel: Combined distribution function of two O‐H RDFs, including ball‐and‐stick representation of structures out of the simulation. Please note that they are further (hydrogen) bonded and do not occur as isolated entities.

As described in the introduction, the solvation of dilute aqueous OH–(aq) alternates between two states.[18] On one hand, a hypercoordinated structure is present with four water hydrogen atoms coordinating to the oxygen atom of hydroxide, while no hydrogen bond is being donated. On the other hand, a tetrahedral configuration forms with up to three water hydrogen atoms coordinating to the oxygen atom of the anion, and water oxygen atom to its hydrogen atom. This is in stark contrast to our hydroflux system, where we find on average less than two (1.8) water molecules donating a hydrogen bond to the hydroxide anion, with an occasional hydrogen bond donation from the hydroxide to a water molecule (17.7% of the time). Under closer scrutiny, the most often occurring number of hydrogen bonds accepted by a single hydroxide from water molecules is also 2 (43.9%), but single (39.8%) and threefold coordination modes (10.8%) also occur, albeit with much lower probability. The aforementioned hypercoordinated system is very rarely observed in the simulation, on average only 0.6% of the time for a single hydroxide. Unlike an ideal Zundel ion for the hydronium ion (described in the introduction), the hydrogen bonding interplay between the hydroxide ion and the water molecule is clearly asymmetric, with a strong difference between a more covalent O–H bond within the water molecule, and a less covalent intermolecular hydrogen bond. This is obviously visible from the O–H radial distribution function (RDF), showing a minimum [and not a maximum] at this distance (at 128 pm, Figure 4 top right, orange). In the course of a proton transfer, however, a transient quasi‐symmetrical structure as previously experimentally observed [28] occurs with an average lifetime of about 0.2 ps (Figure 4 lower panel). As mentioned above, proton transfers are prevalent, which means that the relative energy of this quasi‐symmetrical structure is low, and therefore it can be seldom observed (4.3% of the time). This observation is fully supported by the unusually high *g(r)* value at the corresponding minimum (at 128 pm) of the O–H RDF (Figure 4 top right, orange), which in other, more dilute systems drops to almost zero. Table 2 also lists hydrogen bonds (160 pm) that appear to be contracted compared to a “normal” water hydrogen bond [29], and that may align with the compressed wire model of *Hassanali*.[23]

**TABLE 2:** Location of the maximum/minimum *g*(*r*) distance (*r*_max_/*r*_min_) and the number integral (NI) at *r*_min_. Note, the second number of the NI is given when the reverse coordination is different, and our simulation temperature was 473 K.

Peak	*r*_max_ / pm	*r*_min_ / pm	NI(*r*_min_)
K‐K (1.)	410	508	6.5
K‐K (2.)	564	682	16.2
K‐O (OH^−^) (H_2_O) (H_3_O_2_^−^)	278	454 422 466 450	10.6/5.3 4.1/4.5 5.1/5.6 1.0/5.2
K···H	298	474	18.1/6.0
O···O (OH)–(H_2_O) (H_2_O)–(H_2_O)	260 259 282	318	2.2 1.8 0.5
O–H (1.)	98	128	1.5/1.0
O···H (2.)	160	240	2.7/1.8
H···H (1.)	162	185	0.8
H···H (2.)	208	292	3.5

The solvation of water molecules also fundamentally differs from that known for pure water or dilute aqueous solutions.[29] In the latter systems, each water molecule donates most often two hydrogen bonds and accepts two more, giving a sum neighbor count of 4. However, in the present system water molecules have most often altogether hydrogen bonding 2 or 3 neighbors (43.7% and 27.8%, respectively), with neighbor counts of 0 (1.7%), 1 (16.1%), and 4 (8.7%) also present. The dissection of this data gives the average number of hydrogen bonds donated to a water molecule, which is surprisingly low (0.41), less than a quarter of that in pure water. The solvation of potassium ions is also very different from that reported earlier for dilute systems. In those cases, as mentioned above, the potassium has on average 6.5 neighboring oxygen atoms, [30, 31] while in our case, it was found to be 10.6. Interestingly, this number resembles that of the [6+4] coordination observed for the KOH·H_2_O crystal,[1] in which six O atoms are found in the range from 279 to 294 pm around the K atom, and four more between 327 and 362 pm (Figure 3). The larger distances in the simulations can be attributed to the dynamic rearrangement characteristic of the liquid phase, although K–O and K–K distances are, in general, very similar to those in the crystal phase. Interestingly, for NaOH solutions Hellström and Behler also found^(10.1039/C6CP06547C)^ a slight increase of total coordination numbers at Na^+^ ions in NaOH solutions as the concentration increased from dilute to x_NaOH_ = 0.3, although the changes—with the different cation in this more dilute solution—were much smaller than in the present case.

**FIGURE 5:**
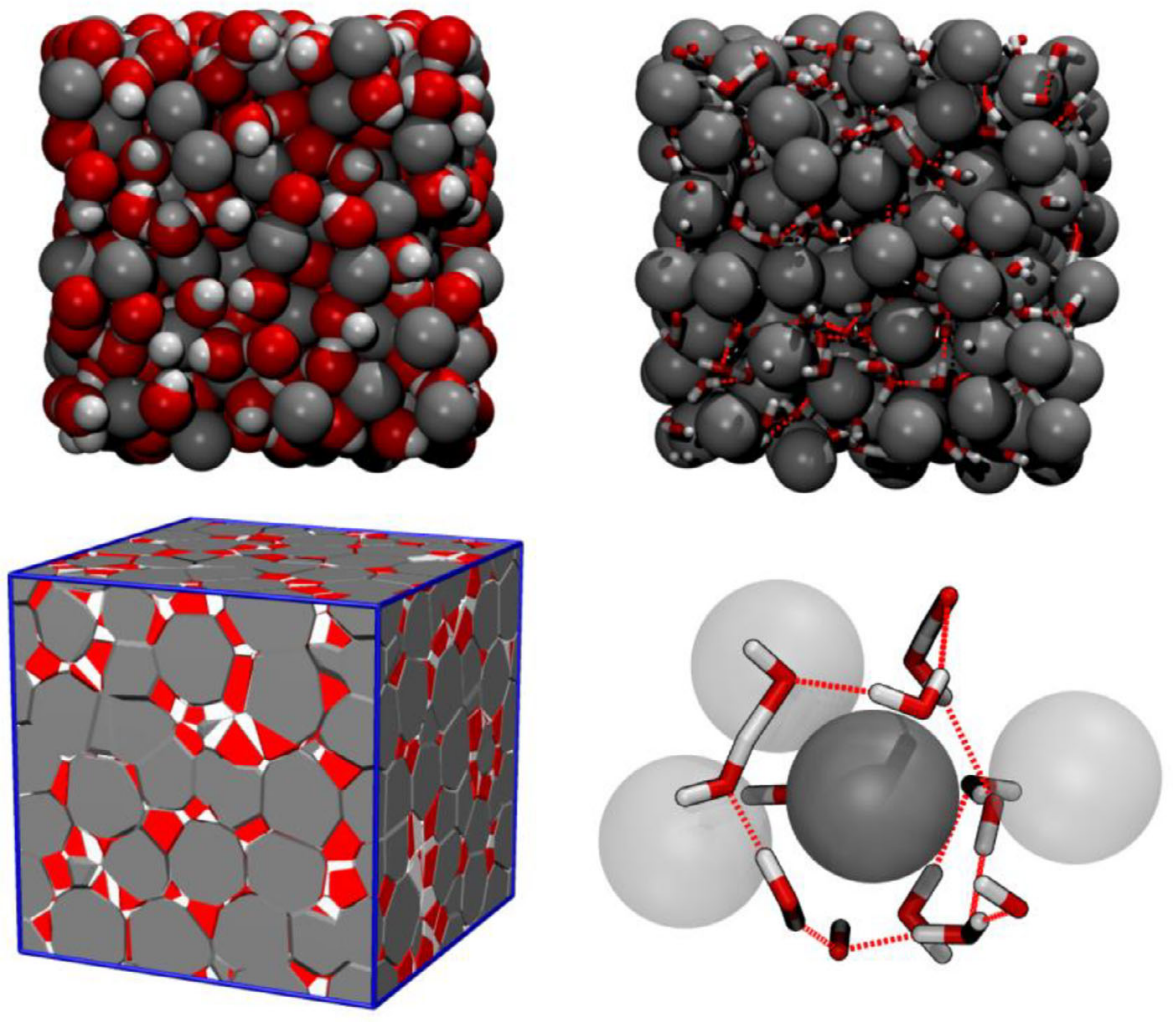
Visualization of the last simulation snapshot. Grey: K atom; Red: oxygen atom; White: Hydrogen atom. Upper left: van der Waals representation; upper right: K atoms as van der Waals representation and O as well as H atoms in Licorice representation. Lower left: The radical Voronoi cell of each atom of the system, colored white for hydrogen atoms, red for oxygen, and grey for potassium. Note, orientation above and below differs. Lower right: Arrangement of potassium cations separated by water molecules and hydroxide ions forming an extended hydrogen bonding network.

Considering the solvation of the three species, as described above, an unusual picture of the overall liquid hydroflux structure emerges. Some water molecules are not participating in the hydrogen bonding network, and their role is solely to coordinate the metal ion. The rest of the water molecules and hydroxide anions mostly form chains (coordination number 2) and sheets (coordination number 3) of hydrogen bonding assemblies, which clearly have termini (coordination number 1), and connections to other chains and sheets (coordination number 3 and 4). This low‐dimensional hydrogen bonding network is also observable visually in the snapshot of the system in Figure 5.

In light of the literature discussions on proton transfer processes in acidic or basic aqueous media, this peculiar structure should have consequences also for the ability of the solution to exhibit structural diffusion. First, the hypercoordinated solvation of the hydroxide does not occur to any significant proportion, and therefore it cannot serve as a resting state for proton transfer processes. Related to this, the reduced proton transfer barriers further facilitate the process, as suggested by the relatively high minimum at 128 pm in the O–H RDF in Figure 4. Second, the reduced dimensionality of the hydrogen bonding network reduces the number of pathways, through which the transferred proton can return to its original location. However, this disruption may also reduce the prevalence of extended hydrogen‐bond chains (percolation pathways) necessary for long‐range proton hopping. Thus, the increased preference of the hydroxide ion to accept a proton from the solution and the reduced barrier for the proton transfer itself, do not necessitate the presence of an actual effective Grotthuss diffusion in the system.

**FIGURE 6:**
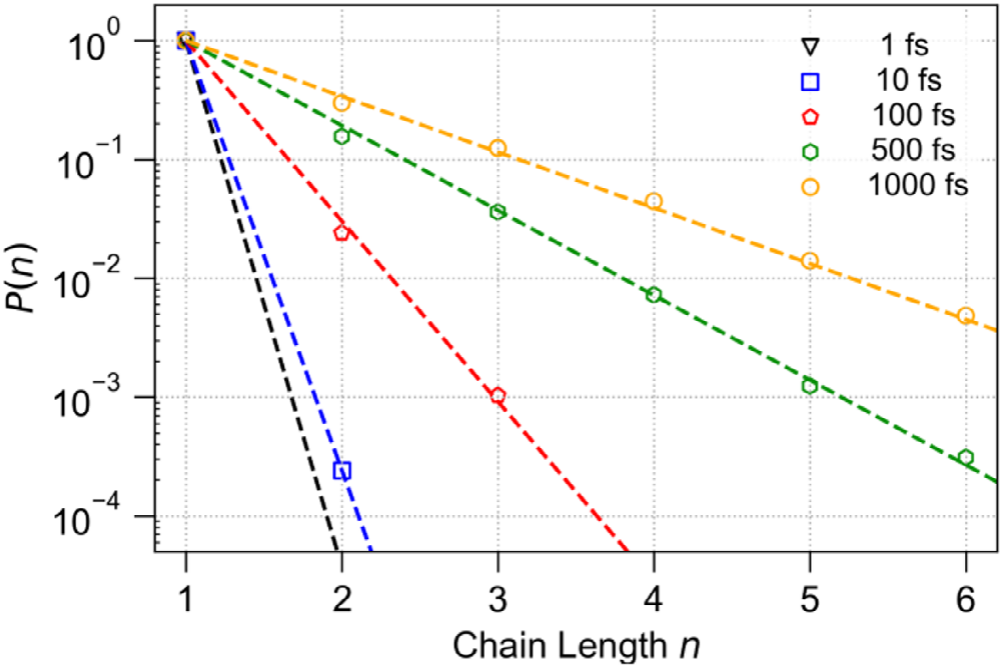
Cumulative percentage *P(n)* of proton transfer events that lead to a chain of *n* correlated proton transfers within a time frame of *τ*  =  1 fs to 1000 fs between each individual proton transfer.

To quantify structural diffusion, we defined the oxygen atom partner of each hydrogen atom at each step of the simulation, to which it is covalently bound. When a proton transfer occurs, a change in this data can be observed. The subsequent proton transfers along a hydrogen bonding chain can be, therefore, defined, and the length of the chain along which the charge moves can be determined as well. Within this framework, we observed proton transfer events in unprecedented chain lengths involving up to seven oxygen atoms, which occurred not more than 1 ps within each other. Characterizing the prevalence of these chain events yields Figure 6 showing their probability within different time limits along different chain lengths. The data show that at least three oxygen atom hops occur within 1 ps of each other in 10% of the cases, whereas 0.4% of the proton transfers lead to chain events of length 6 or longer involving seven distinct oxygen atoms or more. This data clearly shows that there is a significant Grotthuss diffusion in the system.

## 3 Conclusion

This study provides detailed evidence that water undergoes significant denaturation in highly concentrated mixtures with NaOH or KOH. Vapor pressure decreases and boiling points increase with increasing concentrations. A clear change in the slope of this correlation occurs at base concentrations *c*_B_ between 20 and 30 mol L^−1^, marking the transition from aqueous solutions — as used, for example, in hydrothermal syntheses and for electrolytes — to wet salt melts, known as hydroflux media. At molar water‐base ratios of *q*(A) ≤ 1 (*c*_B_ ≥ 55.5 mol L^−1^), the water has a greatly reduced chemical activity and is essentially trapped in the melt by strong bonding interactions. Ab initio molecular‐dynamics simulations reveal that the hydroxide and the water molecules form structural arrangements that differ from the solvation motives in more dilute or pure and ambient systems. Hydroxide ions are not found in a hypercoordinated structure, which hinders mobile protons to move, but are part of wire‐like structures. Similarly, water does not assume the well‐known fourfold coordination. These situations make the hydroflux system predestined for Grotthuss diffusion, which we observe within 1 ps in wires with up to five oxygen atoms.

Overall, this combined experimental and computational analysis highlights the significant effect that ultra‐alkaline conditions have on the macroscopic and microscopic properties of water. The results clearly demonstrate how extreme alkalinity disrupts traditional hydrogen bonding, alters vapor‐liquid equilibria, and significantly affects molecular coordination and proton dynamics. These findings improve our understanding of how water behaves in alkaline salt melts, informing theoretical developments and practical applications in chemistry and materials science involving ultra‐alkaline environments.

## Conflicts of Interest

The authors declare no conflict of interest.

## Supporting information



The Supporting Information provides details and additional data about the device, the pressure measurement methodology, and the AIMD simulations. The authors have cited additional references within the Supporting Information [32, 33, 34, 35, 36, 37, 38, 39, 40, 41, 42, 43, 44, 45, 46, 47].
**Supporting file 1**: chem70521‐sup‐0001‐SuppMat.pdf
